# Treatment of Intrabony Defects with Non-Surgical Subgingival Debridement: A Radiographic Evaluation of Bone Gain Using an Experimental Digital Software “Bone Defect Analysis (BDA)”

**DOI:** 10.3390/jcm13154315

**Published:** 2024-07-24

**Authors:** Alessia Pardo, Laura Bonfante, Annarita Signoriello, Andrea Benetti, Marco Barillari, Piero Zanutto, Giorgio Lombardo

**Affiliations:** 1 Dentistry and Maxillo-Facial Surgery Unit, Department of Surgery, Dentistry, Paediatrics and Gynaecology (DIPSCOMI), University of Verona, Piazzale L.A. Scuro 10, 37134 Verona, Italy; alessia.pardo@univr.it (A.P.); bonfantelaura@yahoo.it (L.B.); abenetti@sorrisodeciso.it (A.B.); giorgio.lombardo@univr.it (G.L.); 2 Radiology Unit, Department of Diagnostics and Public Health, University of Verona, Piazzale L.A. Scuro 10, 37134 Verona, Italy; marco.barillari@aovr.veneto.it (M.B.); piero.zanutto@gmail.com (P.Z.)

**Keywords:** bone defect, bone gain, defect angle, BDA software, minimally invasive, non-surgical, radiographic

## Abstract

**Background:** The aim of this study was to retrospectively evaluate the 3-year radiographic outcomes of periodontal intrabony defects treated with non-surgical subgingival therapy (NST), assessing radiographic bone gain (RBG) through experimental digital software, named “Bone Defect Analysis (BDA)”. **Methods:** The study included 17 intrabony defects in 14 patients. BDA software (version 1) was used on radiographs to calculate RBG (in %) and variations in defect angle (in °) between baseline (T0) and 3-year follow-up (T1). Soft tissue conditions were registered, reporting bleeding on probing (BOP), probing pocket depth (PPD), and clinical attachment level (CAL). Defects were analyzed according to angles less (group A) or greater (group B) than 30°. **Results:** Nine and eight defects were, respectively, analyzed in groups A and B. Three years after treatment, an average RBG of 12.28% was found overall, with 13.25% and 10.11% for groups A and B, respectively (*p* = 0.28). Clinically, a mean CAL of 6.05 mm at T1 (from 10.94 mm at T0) was found, with 6.88 mm and 5.12 mm in groups A and B, respectively (*p* = 0.07). **Conclusions:** BDA software demonstrated predictability in the evaluation of bone variations after NST, revealing better clinical findings for intrabony defects with an initial smaller angle.

## 1. Introduction

Periodontitis, known as a chronic disease predominantly affecting adults and characterized by a bacterial etiology and an inflammatory pathogenesis, leads to progressive destruction of soft and hard tissues essential in ensuring support and tooth stability [1]. Its onset is caused by a dysbiosis of the commensal oral microbiota (dental plaque), which interacts with host immune defenses, leading to widespread inflammation [2]. This pathophysiological mechanism persists through clinical phases of activity and quiescence [3], until, in the worst case, the affected tooth is extracted. Once the microbial biofilm is mechanically removed, in cases of positive response to therapy, the inflammation subsides.

The most representative clinical and radiographical finding of periodontitis due to the destruction of supportive periodontal tissues is the periodontal pocket. Intrabony periodontal defects [4] are defined as defects extending below the bone crest and classified according to the location and number of bony walls. Regenerative treatment of intrabony defects using non-surgical subgingival therapy (NST) [5] demonstrates a reduction in pocket depth and gain in clinical attachment level. While deep defects seem to have an influence only on radiographic bone gain, reduced defect angles and increased number of walls seem to influence both radiographic bone gain and clinical attachment level. The baseline morphology of the defect can thus predict a possible prognosis after regenerative therapy [5,6,7]. 

A precise and correct assessment of periodontal defects constitutes a fundamental aspect in therapeutic planning. Based on current evidence [8,9], clinicians should consider the significant limitations inherently associated with intraoral radiographs. Despite radiographic examinations usually offering an objective and standardized mean of assessing the condition of hard tissues [8], it must be underlined that, generally, consistent bone loss needs to occur to become visible on a radiographic level. As two-dimensional (2D) images represent three-dimensional objects, consequent superimposition of anatomical structures possibly leads to less precise rendering of details [10]. Measurement of alveolar bone loss with 2D images is confined to the parasagittal plane, which may not fully capture the complete osseous architecture of the alveolar bone ridge [11]. Furthermore, this assessment of bone defects does not provide a complete view of the bucco-lingual aspects of the interproximal bone. For this reason, radiographic tests seem to provide high positive predictability if the defect is visible (as it is likely that it is actually present), but low negative predictability if the defect is not visible (as it does not mean that it is not present). It is reported that early lesions are often difficult to diagnose accurately since radiographic evidence appears only after 30–50% of bone resorption [12].

While some authors [9,13,14,15] declared a tendency to underestimate alveolar bone loss, another study [15] reported an extreme accuracy in identifying bone defects. In this proposal, studies in the literature regarding the treatment of periodontal bone defects with non-surgical therapy do not present homogeneous designs and methodologies. Some authors [5,16,17] conducted randomized trials, reporting the use of semi-automated radiographic software with specific landmarks [5] or not reporting any information for bone levels calculation [16]; some others [18,19] proposed retrospective [19,20] or prospective long-term [18] evaluations again using landmarks and software-assisted analysis of images; and another randomized trial [21] focused more on clinical parameters rather than radiographic assessment.

Considering the great importance of high-quality images for adequate interpretation and measurement of both quantitative and qualitative bone variations [22], easily applicable methods of evaluation are encouraged independently from the radiographic system used, allowing accurate and predictable measurements. In the light of these considerations, the aim of this retrospective study was to test the effectiveness of an experimental software, named “Bone Defect Analysis (BDA),” which can assist operators in detecting and consequently calculating radiographic bone gain (RBG) in terms of percentage. A secondary aim was to analyze the radiographic response of intrabony defects 3 years after NST, relating the values of initial defect morphology to final outcomes both for bone gain and clinical attachment level (CAL).

## 2. Materials and Methods

### 2.1. Study Design and Inclusion Criteria

The present retrospective study was designed and conducted in compliance with the principles of the Declaration of Helsinki on medical protocols and ethics and good clinical practice guidelines for research on human beings. Ethical approval was obtained from the University of Verona Institutional Review Board. The nature and aim of the study, together with the anonymity in the scientific use of data, were clearly explained in a written, informative consent form, which was signed by every patient. 

The retrospective study was conducted between January 2024 and March 2024 on available medical records with a 3-year follow-up that satisfied the following inclusion criteria. Subjects included in the study (consecutively treated with NST in 2019 by a single operator) presented:

- American Society of Anesthesiology (ASA) classification status I (that is, negative history for systemic diseases and known drug therapies);
- Diagnosis of stage III or IV periodontitis (grades A to C) [5];
- Presence of ≥1 vertical three-wall “intrabony defect” (PPD > 5 mm with depth of intraosseous defect ≥2 mm on X-ray) and presence of BOP.

Patients who received systemic antibiotic therapy within 3 months, self-proclaimed pregnancy or breastfeeding, and underwent periodontal treatment in the last 12 months were excluded from the study. 

Regarding sample size calculation, a post hoc analysis of power (based on PPD as the main outcome variable) found 99% power for within-group comparisons, both overall and for each group.

### 2.2. NST Therapy

NST was performed with manual (Gracey micro-mini curettes) and mechanical (piezoelectric and/or magnetostrictive) instruments [23]. The minimally invasive protocol used for NST [5,6,18] requires that sites designated to receive surgical treatment undergo accurate scaling and root planning using mini-curettes and ultrasonic devices with specific, thin and delicate tips. These tools were carefully inserted into the periodontal pocket of the tooth associated with the defect to reach the surface of the root and proceed with debridement. Care was taken to preserve the stability and appearance of soft tissues. 

In addition, Helbo PDT photodynamic therapy, which consists of a dye (1% of phenothiazone chloride) and a light source (Helbo TheraLite Laser, Senden, Germany, 660 nm diode laser, Bredent) was used [24]. The treatment consisted of staining the site with 1% phenothiazine chloride for 3 s, washing the excess dye with saline, and irradiation of each treated tooth for one second. Treatment with photodynamic therapy was carried out before NST to reduce inflammation and after treatment to reduce plaque and bio-stimulate tissues. 

### 2.3. Soft Tissues Assessment

A periodontal probe (Florida Probe; Florida Probes Company, Gainesville, FL, USA) was used to assess periodontal soft tissues at T0 and T1 in terms of [18]: PPD (measured in mm as the distance between the gingival margin and the base of the periodontal pocket), CAL (measured in mm as the distance from the CEJ to the location of the probe tip), and BOP (measured as 0 (no bleeding) or 1 (bleeding), recorded after probing for PPD).

### 2.4. BDA Software

BDA software is a visualization tool capable of assisting the operator in calculating the angle of intrabony defects and the percentage of RBG on radiographic images, whether they are obtained with analog or digital systems and with or without the same execution geometry between X-ray source and detector. A comparison was thus performed between two radiographic images, at baseline T0 (treatment) and after 3 years T1 (3-year follow-up). 

BDA was created using Python 3.7 scripting language and ported using Pyinstaller. The images were created using open computer vision (OpenCV) libraries and processed in jpg format. By marking landmarks on the radiograph, the software is able to define the percentage of root surface covered by bone tissue. Percentages can be compared between pre- and post-treatment radiographs of the same dental element without the aid of the same geometry of execution of the radiographs. The use of a correction factor isn’t necessary and made possible by the use of percentages to define the long axis of the dental element. When we use percentages, we are scaling all values to a reference [25]. For the first image (at baseline), the root surface covered by bone tissue is scaled to the length of the tooth in the baseline radiograph; in the second image (at follow-up), the root surface covered by bone tissue is scaled to the length of the tooth in the follow-up image. As both images have the same tooth subject, the real length of the tooth is the same. The fact that two images have different pixel dimensions for the dental element is a matter of image magnification [26] caused by the geometry between the X-ray source and the detector at the time the X-ray is performed. We therefore solve the magnification problem by defining the long axis of the dental element in percentage because the tooth is the same in both radiographs.

**Figure 1:**
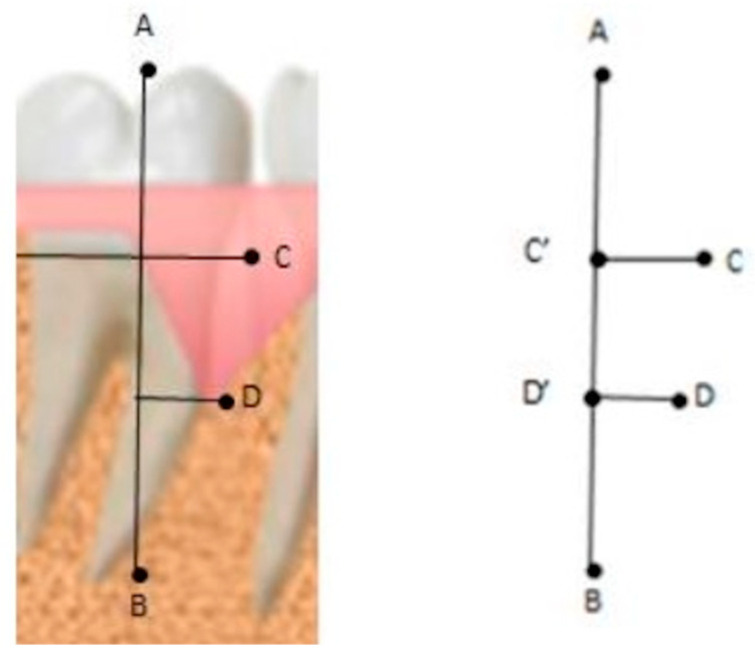
RSA in percentage is the ratio between segments D’B and C’B. C’ and D’ are obtained by projecting C and D on the line AB, defined by points A (coronal margin) and B (root apex). C’B thus corresponds to the length of the root and D’B to the length of the root completely covered by bone tissue.

RBG can be calculated as the variation between T0 and T1, in terms of percentage, of the root surface covered by bone tissue. Through the software, the user can first indicate four points (see Figure 1):

- Point A: coronal margin;
- Point B: root apex;
- Point C: enamel–cement junction (CEJ) of the tooth involved in the intraosseous defect (constant reference point), which can be identified in the radiographic image by the difference in radiopacity of the enamel compared to the cement or by their variation in intensity since the enamel is radiopaque compared to the cement. If restorations are present, the apical margin of the restoration is used to replace the CEJ as a fixed reference point;
- Point D: the most apical point of the angular defect (bottom of the defect).

The percentage of the root surface area covered by bone (radiographic surface area, RSA, in %) is approximated as the ratio D’B/C’B, where D’ and C’ are the projections of points D and C on the segment AB. The remaining percentage corresponds to bone loss. Comparing RSA (radiographic surface area) at T0 with RSA at T1 (in %) yields the final RBG, which can be obtained in %. 

**Figure 2:**
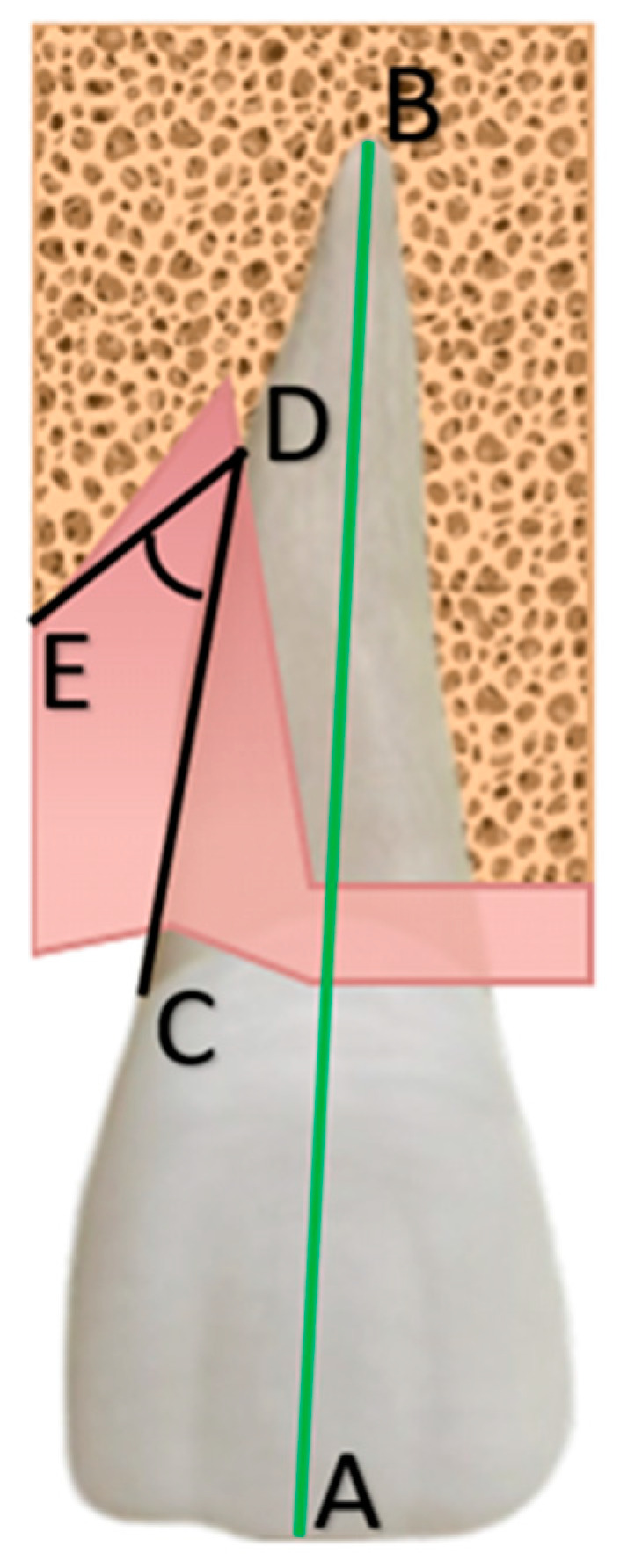
The angle at the base of the intrabony defect (in black) is defined by two segments: respectively, CD, which represents the surface of the involved tooth, and ED, which represents the surface of the bone defect; AB (green line) represents the distance between coronal margin and root apex.

Furthermore, point E (see Figure 2) is fixed as the most coronal point of the angular intrabony defect. Connecting it with points C and D, it is possible to detect the angle at the base of the intrabony defect.

**Figure 3:**
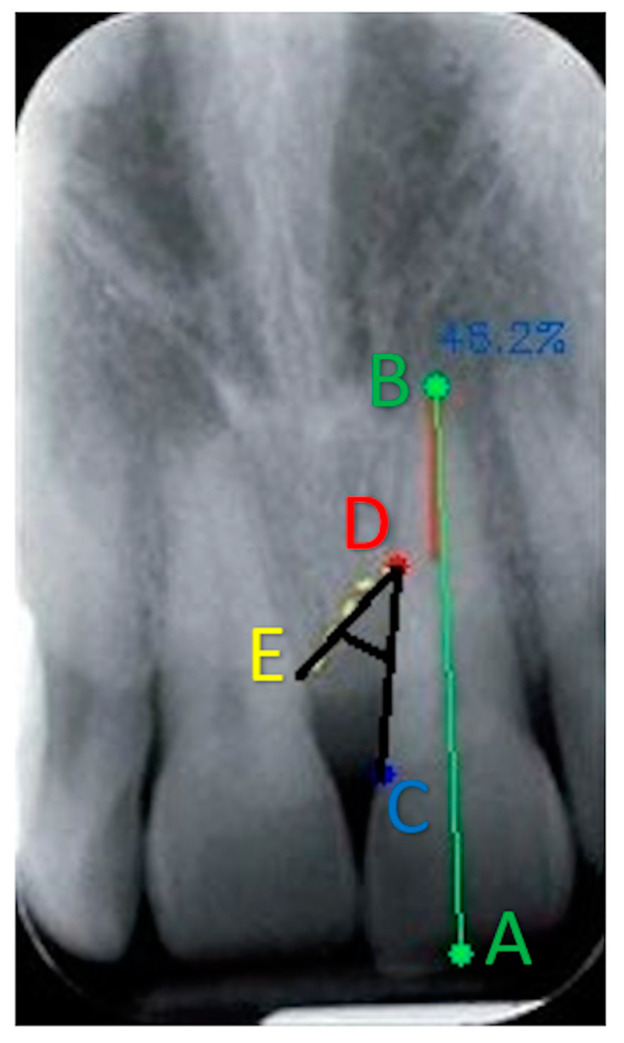
From the software calculation, the mesial surface of the root completely covered by bone tissue is equal to 46.2%, which means that the bone loss is approximately equal to 54%. Points A and B are connected by the green line; points C, D, and E are, respectively, visible as blue, red, and yellow indicators. By selecting point E and connecting it with points C and D, it is possible to detect the angle at the base of the intrabony defect (in black).

Using BDA on a radiographic image, as shown in Figure 3, points A and B are connected by the green line, point C is visible as a blue indicator, and point D as a red indicator. The defect component is also measured by adding point E (yellow indicator).

To verify whether the extent of the intrabony defect angle had an influence on the final RBG, defects were analyzed according to angles less (group A) or greater (group B) than 30°.

To test the effectiveness of BDA software and to analyze in detail the outcomes of RBG (in %) three years after NST, linear measurements of projections of DB and CB on standardized radiographs were assessed with the aid of the widely employed software program ImageJ (Rasband, W.S., ImageJ, U. S. National Institutes of Health, Bethesda, MD, USA, version 1.54h) [27], which uses a measuring tool in conjunction with a magnification tool. To correct the distortion of the radiographic image, the apparent size of each linear segment (measured directly on the radiograph) was compared with values of crown/root lengths known in the literature (used as reference scale) to determine, with adequate precision, the amount of any changes in the bone for each defect. The measurements were made to the nearest 0.01 mm, converted to %, and compared with measurements obtained from BDA software.

An evaluation of these same defects on standardized radiographs was performed with a known digital software (Image J), which can be considered a sort of “control group” in terms of retrospective comparison, to test the effectiveness of the experimental BDA software. 

Both for measures with BDA software and ImageJ software, one dentist who was not involved in the treatment of the patients completed all of the measurements on periapical radiographs; the observation intervals of the radiographs were masked to the examiner. Before the start of the study, this investigator was calibrated for adequate intra-examiner levels of accuracy and reproducibility in recording radiographic parameters. Three radiographs were utilized for this purpose, and duplicate measurements for D’B and C’B were collected with an interval of 24 h between the first and second recordings. The intra-class correlation coefficients, used as a measure of intra-examiner reproducibility, had to be greater than 0.8.

### 2.5. Statistical Analysis

All data analysis was carried out using Stata v.13.0 for Macintosh (StataCorp, College Station, TX, USA). The normality assumptions for continuous data were assessed using the Shapiro–Wilk test; mean and standard deviations were reported for normally distributed data, median and interquartile range (IQR) otherwise. Absolute frequencies, percentages, and 95% confidence intervals were reported for categorical data. The comparison between the means of continuous variables at different times (T0 and T1) was performed using the paired Student’s *t*-test or Wilcoxon matched-pairs signed-rank test. The comparison between the means of groups A and B was performed with the unpaired Student’s *t*-test or Wilcoxon rank-sum test. The significance level was set at 0.05. The study was conducted according to STROBE checklist guidelines [28]. 

## 3. Results

The clinical sample consisted of 17 consecutively treated intrabony defects in 14 patients treated with minimally invasive NST. Treatment with photodynamic therapy was carried out for nine defects. Periodontal defects presented as stage III in eight cases and stage IV in nine cases; grade A in two cases, grade B in seven cases, and grade C in eight cases.

Demographics are reported in Table 1.

The mean values for PPD and CAL were registered at T0 and T1 (see Table 2 and Table 3). Furthermore, mobility registered for all cases at T0 was still present in only five cases at T1.

The overall value of BOP decreased from 100% (all 17 defects) at T0 to 29.41% (5 defects) at T1.

Variations in defect angles are reported in Table 4.

Three years after treatment, an average RBG of 12.28% was found overall, with 13.25% and 10.11% for groups A and B, respectively (see Table 5). Regarding the comparison of average values of RBG in % between groups, no significant differences were observed (*p* = 0.28), meaning that the angle of the defect did not significantly influence the treatment outcome. Variations in RBG found with BDA are reported in Table 5.

Values of RBG in mm were also assessed as linear measurements on the same radiographic images with ImageJ software, at T0 and at T1, with statistically significant differences between time intervals (*p* = 0.001). Expressing these values in percentages (see Table 6), the comparison with outcomes found with BDA software showed that the results were superimposable: an average RBG of 10.51% was found overall, with 11.07% and 10.51% for groups A and B, respectively (*p* = 0.67).

## 4. Clinical Cases

**Figure 4:**
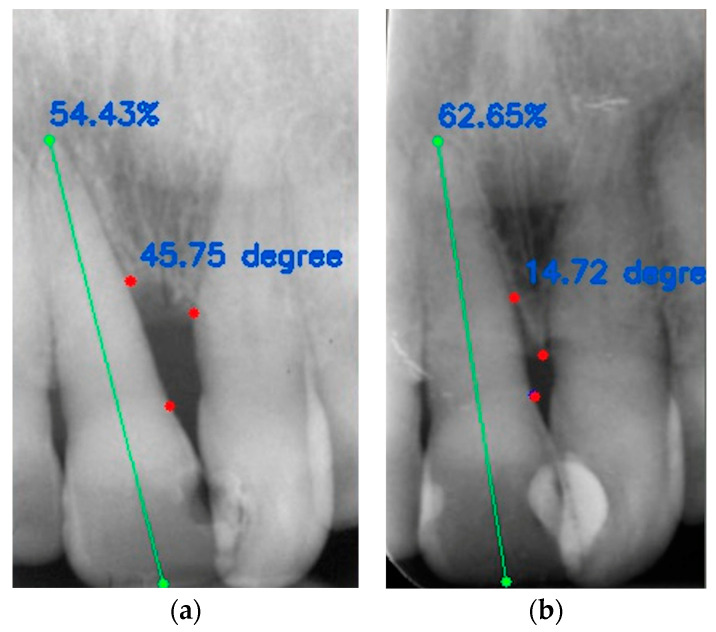
Radiographic case of a bone defect between the mesial surface of element 1.1 and the mesial surface of element 2.1: (**a**) angle at T0 is 45.75° and RSA at T0 is 54.43%; (**b**) angle at T1 is 14.72° and RSA at T1 is 62.65%. The green line represents the distance between coronal margin and root apex. The red dots represent the angle at the base of the intrabony defect, respectively at T0 and at T1.

**Figure 5:**
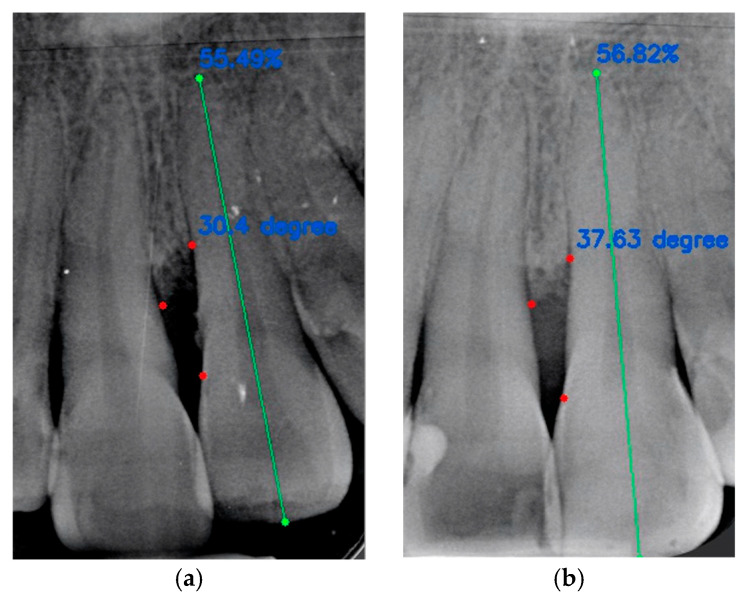
Radiographic case of a bone defect between the mesial surface of element 1.1 and the mesial surface of element 2.1: (**a**) angle at T0 is 30.4° and RSA at T0 is 55.49%; (**b**) angle at T1 is 37.63° and RSA at T1 is 56.82%. The green line represents the distance between coronal margin and root apex. The red dots represent the angle at the base of the intrabony defect, respectively at T0 and at T1.

Examples of cases analyzed are visible in Figure 4a,b and Figure 5a,b.

## 5. Discussion

This study aimed to test the functionality and feasibility of a new experimental software, BDA, in assisting clinicians in the evaluation of intrabony defect angles [29]. With regard to the present analysis, BDA was found to be useful in dentistry for the calculation of the percentage of bone loss at baseline and the percentage of radiographic bone gain after non-surgical periodontal treatment, simply by marking defined landmarks and points on the radiographs. The proposed software thus allowed an accurate evaluation of the radiographic angle as a predictive variable for the assessment of the intraosseous bony defect component [17], enabling a precise analysis of percentages of bone variation between baseline and follow-up.

The issue of different pixel dimensions for a tooth in two radiographs is a matter of image magnification [25,26] caused by the geometry between the X-ray source and the detector at the time of X-ray execution. The advantage of BDA software is assisting the operator in performing measurements of the angle of intrabony defects and the percentage of RBG on radiographic images available for any system, without the aid of the same geometry of execution of the radiographs. Furthermore, the magnification problem is solved by defining the long axis of the dental element in percentage (corrective factor) because the tooth is the same in both radiographs.

In addition to the proposed BDA, another technique introduced in 1930 to evaluate bone and tissue gain is digital subtraction radiography (DSR) [30,31,32]. Its main advantage is the ability to observe small changes in the order of 5% of mineral loss, while in subjective analysis, the loss from about 30% to 60% of volume is usually required to see some changes [33]. This advantage justifies the use of DSR in dentistry, especially in implantology and periodontology, for monitoring the progression of lesions in alveolar bone tissue or implant osseointegration. Therefore, it is possible to observe an increase in bone density and to evaluate X-ray variations in intrabony defects through the radiopacity given by the remineralization of cortical bone [25,34]. Subtracting two X-ray images means superimposing these images to analyze the differences that occurred between them over time, and even small differences can be seen through this technique [35,36]. Differently from DSR, in this study, the recording of the image a priori was not carried out with BDA because we tried to recreate a situation as similar as possible to that which usually occurs. This means that the X-rays were taken using positioners but without bite recording, and no devices were used for the geometric alignment of objects, detectors, and X-ray beams. 

In addition, to test the effectiveness of BDA software and to analyze in detail the outcomes of RBG (in %) three years after NST, linear measurements of projections of DB and CB were assessed with the aid of the widely employed software program ImageJ. The comparison between RBG values found with BDA and with ImageJ were superimposable, as an average RBG of 10.51% was found overall, with 11.07% and 10.51% for groups A and B, respectively (*p* = 0.67). As the aim of this study was to test BDA in assessing the radiographic response of intrabony defects 3 years after non-surgical therapy, relating the values of the initial defect morphology to the final outcomes for bone gain can be relevant in facilitating clinicians to make precise evaluations of bone gain in daily clinical practice, apart from the radiographic system used.

Risk of disease progression, in terms of residual periodontal pockets during supportive periodontal treatment following cause-related therapy, usually represents a requirement for further surgical treatment [37]. Historically, intrabony lesions were initially treated with resective surgical techniques that sought to remove them [38]. As time went on, among possible surgical treatment modalities, periodontal regeneration aimed to restore lost periodontal tissues in terms of PPD reduction, CAL gain, and bone fill [39], showing higher benefits compared with access flaps in term of clinical and radiographic parameters [40]. Minimally invasive surgery (MIS) was proposed in conjunction with biomaterials, e.g., demineralized freeze-dried bone allograft (DFDBA) and vicryl mesh membranes [41]. In this proposal, the minimally invasive surgical technique (MIST) [42] was introduced as a modification of previous flap designs with papilla preservation [43,44], followed by a single-flap approach (SFA) [45] and double-flap approach (DFA) in the case of complex intrabony defects.

On the other hand, to eliminate the necessity for surgical procedures in the treatment of intrabony defects, minimally invasive non-surgical therapy (MINST) [21] was hypothesized as a non-surgical approach with the use of magnification for better visualization and the application of less invasive instruments for minimal tissue trauma, revealing overall favorable results for the treatment of intrabony defects and avoiding, at the same time, the psychological burden on patients, surgical morbidity, or esthetic complications. 

In a retrospective 1-year analysis [19], the average PPD and CAL reductions in the intrabony defects were 3.12 mm and 2.78 mm, respectively, and the average radiographic intrabony vertical defect depth was reduced by 2.93 mm (from 6.74 mm to 3.81 mm), whereas the average defect angle changed from 28.5° at baseline to 44.4° at re-evaluation. In a long-term study [18], 14 individuals with 17 intrabony abnormalities were included, and no substantial differences were reported from 1 to 5 years, finding PPD, CAL, and radiographic intrabony vertical defect depth reductions of 3.6, 3.5, and 2.6 mm, respectively, 5 years after treatment. 

Defect morphology has a significant impact on periodontal healing after regenerative treatment of intrabony defects. This was established in studies [46,47,48,49] that found the depth of the intrabony component of the defect influenced the amount of clinical attachment and bone gain after 1 year, and the deeper the defect, the greater was the amount of clinical improvement. Deep and shallow lesions, however, were shown to have the “same potential” for regeneration in a multicenter controlled study [50]. In other words, after treating deep defects, we would anticipate greater linear gains in attachment than after treating shallow defects, but both defects can express regenerative potential up to the full resolution of the intrabony component of the defect. The defect width of the intrabony component, which is determined by the angle formed by the bony wall and the long axis of the tooth [51], is another significant morphological feature. Again, reduced clinical attachment levels and bone gain at 1 year were linked to wider defects.

In this study, 3 years after treatment, an average RBG of 12.28% was found overall, with 13.25% and 10.11% for groups A and B, respectively. Regarding the comparison of average values of RBG in % between groups, no significant differences were observed (*p* = 0.28), meaning that the angle of the defect did not influence the treatment outcome. Even if intrabony defects with angles less than 30 degrees had greater and constant RBG compared to the group with angles greater than 30 degrees, the small number of defects examined in this study probably influenced the outcomes.

The methodological approach used in this study, consisting of the use of manual and mechanical instruments, is in line with other current developments in periodontal strategies for NST, which mainly focus on the employment of means or substances (e.g., ozone, laser, hyperbaric oxygen therapy, topical agents) able to disaggregate the biofilm [52,53]. In this proposal, photodynamic therapy [24], applied only in those cases which were particularly characterized by acute gingival inflammation and profuse bleeding, must be considered an additional means of decreasing signs of inflammation (overall value of BOP decreased from 100% at T0 to 29.41% at T1) without any implication on bone gain, which was instead the main outcome of the study.

Finally, as in regenerative periodontal surgery, the wider the angle, the smaller the amount of regeneration. The study tested, in terms of verification of potential RBG through the use of BDA, whether a prediction of the amplitude of a radiographic defect angle at baseline [17] can improve the extent and predictability of regeneration after non-surgical periodontal therapy at follow-up. 

The results of the present study, the limitations of which consist of a retrospective evaluation of a small number of defects, should be validated by further prospective investigations with longer follow-ups, more cases, and with an empowerment of the potential of BDA functionalities, also associating radiographic measurements with the evaluation of all clinical parameters.

## 6. Conclusions

BDA software offers precise measurements of the width of an angle on radiographic images, assuming this variable as predictive for the evaluation of periodontal healing, in terms of RBG, of intrabony defects following non-surgical periodontal therapy. Intrabony defects with a smaller angle of amplitude demonstrated a greater percentage of RBG. BDA software is thus a valid instrument to support clinicians in non-surgical periodontal therapy.

## Figures and Tables

**Table 1:** Patients and tooth characteristics at baseline; variables are expressed as n (%).

Defect Characteristics	n	%
*Sex*		
male	4	23.53
female	13	76.47
*Age* ^1^	53.94 ± 9.62	years
*Defect angle*		
<30° (group A)	9	57.94
>30° (group B)	8	47.06
*Tooth*		
central incisor	5	29.41
lateral incisor	3	17.64
canine	2	11.76
first premolar	0	0
second premolar	2	11.76
first molar	5	29.41
second molar	0	0

^1^ Age is presented as mean (±standard deviation).

**Table 2:** PPD values are presented in mm as mean (± standard deviation).

PPD	T0	T1	∆(T0 − T1)	*p* Value
OVERALL	9.76 ± 2.48	3.76 ± 0.83	6 ± 2.2	0.001 *
GROUP A	11.11 ± 2.2	4.11 ± 0.92	7 ± 2.29	0.001 *
GROUP B	8.25 ± 1.9	3.37 ± 0.51	4.87 ± 1.55	0.001 *
*p* value	0.01 *	0.06	0.04 *	

* statistically significant differences between groups/observation times (T0 = baseline; T1 = 3-year follow-up).

**Table 3:** CAL values are presented in mm as mean (± standard deviation).

CAL	T0	T1	∆(T0 − T1)	*p* Value
OVERALL	10.94 ± 2.79	6.05 ± 2.07	4.88 ± 1.76	0.001 *
GROUP A	12.44 ± 2.06	6.88 ± 1.76	5.55 ± 1.94	0.001 *
GROUP B	9.25 ± 2.6	5.12 ± 2.1	4.12 ± 1.24	0.001 *
*p* value	0.01 *	0.07	0.09	

* statistically significant differences between groups/observation times (T0 = baseline; T1 = 3-year follow-up).

**Table 4:** Defect angle values are presented in °; values are presented as median (IQR).

		T0	T1	∆(T0 − T1)	*p* Value
ANGLE	OVERALL	28.73 (12.56)	37.63 (21.6)	9.18 (14.49)	0.16
	GROUP A	24.33 (4.48)	28.6 (18.36)	10.09 (12.7)	0.02 *
	GROUP B	37.31 (5.05)	38.07 (29.86)	3.75 (38.14)	0.77
	*p* value	0.001 *	0.84	0.21	

* statistically significant differences between groups/observation times (T0 = baseline; T1 = 3-year follow-up).

**Table 5:** RBG values from BDA are presented in %; values are presented as median (IQR).

BONE VARIATION	RSA T0	RSA T1	RBG	*p* Value
OVERALL	42.19 (22.37)	56.82 (25.12)	12.28 (10.94)	0.001 *
GROUP A	39.41 (25.69)	50.89 (24.88)	13.25 (13.17)	0.007 *
GROUP B	53.4 (22.41)	59.34 (12.01)	10.11 (10.53)	0.01 *
*p* value	0.21	0.44	0.28	

* statistically significant differences between groups/observation times (T0 = baseline; T1 = 3-year follow-up).

**Table 6:** RBG values from ImageJ are presented in %; values are presented as median (IQR).

		RSA T0	RSA T1	RBG	*p* Value
BONE VARIATION	OVERALL	41.49 (24.11)	55.53 (23.72)	10.51 (9.09)	0.001 *
	GROUP A	37.61 (29.12)	44.48 (22.69)	11.07 (11.95)	0.01 *
	GROUP B	53.27 (21.36)	59 (11.35)	10.51 (9.91)	0.01 *
	*p* value	0.09	0.24	0.67	

* statistically significant differences between groups/observation times (T0 = baseline; T1 = 3-year follow-up).

## Data Availability

The data presented in this study are available upon request from the corresponding author.
